# Characterizing Component Activities of Older Adult Sedentary Time by Age, Gender, and Device-Based Sitting Patterns

**DOI:** 10.1093/geroni/igab046.1311

**Published:** 2021-12-17

**Authors:** Mikael Anne Greenwood-Hickman, Rod Walker, John Bellettiere, Andrea LaCroix, Dori Rosenberg

**Affiliations:** 1 Kaiser Permanente Washington, Seattle, Washington, United States; 2 Kaiser Permanente Washington Health Research Institute, Seattle, Washington, United States; 3 University of California, San Diego Herbert Wertheim School of Public Health and Human Longevity Science, La Jolla, California, United States

## Abstract

The activities that compose older adults’ considerable sedentary time are not well characterized. We described daily time spent in self-reported sedentary activities and explored differences by age, gender, and activPAL sitting patterns. Participants self-reported a total of 10.7 hours of sitting time and spent the most time watching TV (2.6 hrs/day), using the computer (1.7 hrs/day), and reading (1.6 hrs/day). Women spent more time watching TV, engaged in hobbies, and socializing and less time on the computer compared to men. Older participants spent more time watching TV, reading, and participating in group activities and less time on the computer than younger participants. Those with low activPAL sitting time and frequent activPAL sitting breaks (low mean bout duration) ~1 hr /day less watching TV than those with high activPAL sitting time. These findings help illuminate future intervention targets and lay the path to explore associations between different sedentary activities and health.

